# Application of Evans Index in Normal Pressure Hydrocephalus Patients: A Mini Review

**DOI:** 10.3389/fnagi.2021.783092

**Published:** 2022-01-11

**Authors:** Xi Zhou, Jun Xia

**Affiliations:** Department of Radiology, The First Affiliated Hospital of Shenzhen University, Health Science Center, Shenzhen University, Shenzhen Second People's Hospital, Shenzhen, China

**Keywords:** Evans index, ventricular volume, computed tomography, magnetic resonance imaging, normal pressure hydrocephalus

## Abstract

With an ever-growing aging population, the prevalence of normal pressure hydrocephalus (NPH) is increasing. Clinical symptoms of NPH include cognitive impairment, gait disturbance, and urinary incontinence. Surgery can improve symptoms, which leads to the disease's alternative name: treatable dementia. The Evans index (EI), defined as the ratio of the maximal width of the frontal horns to the maximum inner skull diameter, is the most commonly used index to indirectly assess the condition of the ventricles in NPH patients. EI measurement is simple, fast, and does not require any special software; in clinical practice, an EI >0.3 is the criterion for ventricular enlargement. However, EI's measurement methods, threshold setting, correlation with ventricle volume, and even its clinical value has been questioned. Based on the EI, the z-EI and anteroposterior diameter of the lateral ventricle index were derived and are discussed in this review.

## Introduction

Normal pressure hydrocephalus (NPH) refers to a condition in which the cerebrospinal fluid (CSF) pressure is normal, but the hydrocephalus in the ventricular system is dilated. Its clinical manifestations include gait disorders, cognitive disorders, and urinary incontinence (Adams et al., 1965). However, as more than 80% of patients can be treated with surgery to improve their symptoms, including the improvement of cognitive impairment (Jaraj et al., 2016), it is also known as treatable dementia (Nakajima et al., 2021). It mostly affects the elderly (Zaccaria et al., 2020), and according to two recent epidemiological surveys in Sweden (Jaraj et al., 2014; Andersson et al., 2019), the prevalence of NPH among 65-year-olds was 3.7%. More importantly, in these two studies, the prevalence of NPH disease in elderly people over 80 years old was as high as 5.9% and 8.9%. This means that the prevalence of NPH increases with age.

The Evans index (EI) is defined as the ratio of the maximal width of the frontal horns to the maximum inner skull diameter. First proposed by Evans in 1942, it has been used to indirectly assess the expansion of the ventricular system in encephalography (Evans, 1942). Evans retrospectively analyzed the 53 encephalograms of normal patients done at the Children's Hospital of Michigan and Harper Hospital and concluded that the ratio of the transverse diameter of the anterior horns to the internal diameter of the skull could be used as an index to evaluate ventricle size. More importantly, this study showed that the normal ratio lies between 0.20 and 0.25, a ratio between 0.25 and 0.30 represents early or questionable enlargement, and values >0.30 represent definite ventricular enlargement. Now, the EI is not only applicable to computed tomography and magnetic resonance imaging images (Ambarki et al., 2010) but also the most common indirect evaluation method for ventricular system expansion in neurosurgery (He et al., 2020).

The increasing and generalized aging in worldwide societies is expected to result in neurodegenerative diseases related to dementia becoming a serious medical and social problem (Szczepek et al., 2015). NPH is a treatable but underdiagnosed disease. Since early treatment can increase the likelihood of a good outcome (Andren et al., 2014), the correct diagnosis of NPH is of great significance (Jaraj et al., 2017a). In the guidelines for the diagnosis of NPH, an EI >0.30 is the standard for measuring hydrocephalus expansion in the ventricular system (Relkin et al., 2005; Mori et al., 2012; Nakajima et al., 2021). However, several studies have questioned the measurement methods, threshold setting, correlation with ventricular volume (VV), and clinical value of EI, while other studies proposed alternative indices for EI. Therefore, this article aimed to review the latest research on the application of the EI in patients with NPH.

## Methods

A literature search was performed by using the following search terms in different combinations: normal pressure hydrocephalus, Evans index, and ventricular volume. These were searched using four databases: PubMed, Scopus, Google Scholar, and Web of Science. The last search was conducted on September 1, 2021.

The exclusion criteria for the articles were as follows:

(1) Published in a language other than English
(2) Without peer review
(3) Animal model or theoretical articles
(4) Sample size of <10 patients.

The inclusion criteria for the article were as follows:

(1) Background introduction of NPH and EI
(2) EI measurement method
(3) Threshold setting of the EI
(4) Correlation between the EI and VV
(5) Clinical value of the EI
(6) Alternative index to the EI.

Considering that some articles may discuss multiple aspects mentioned above at the same time, we decided to classify it into only one main discussion aspect.

## Results

**Figure 1:**
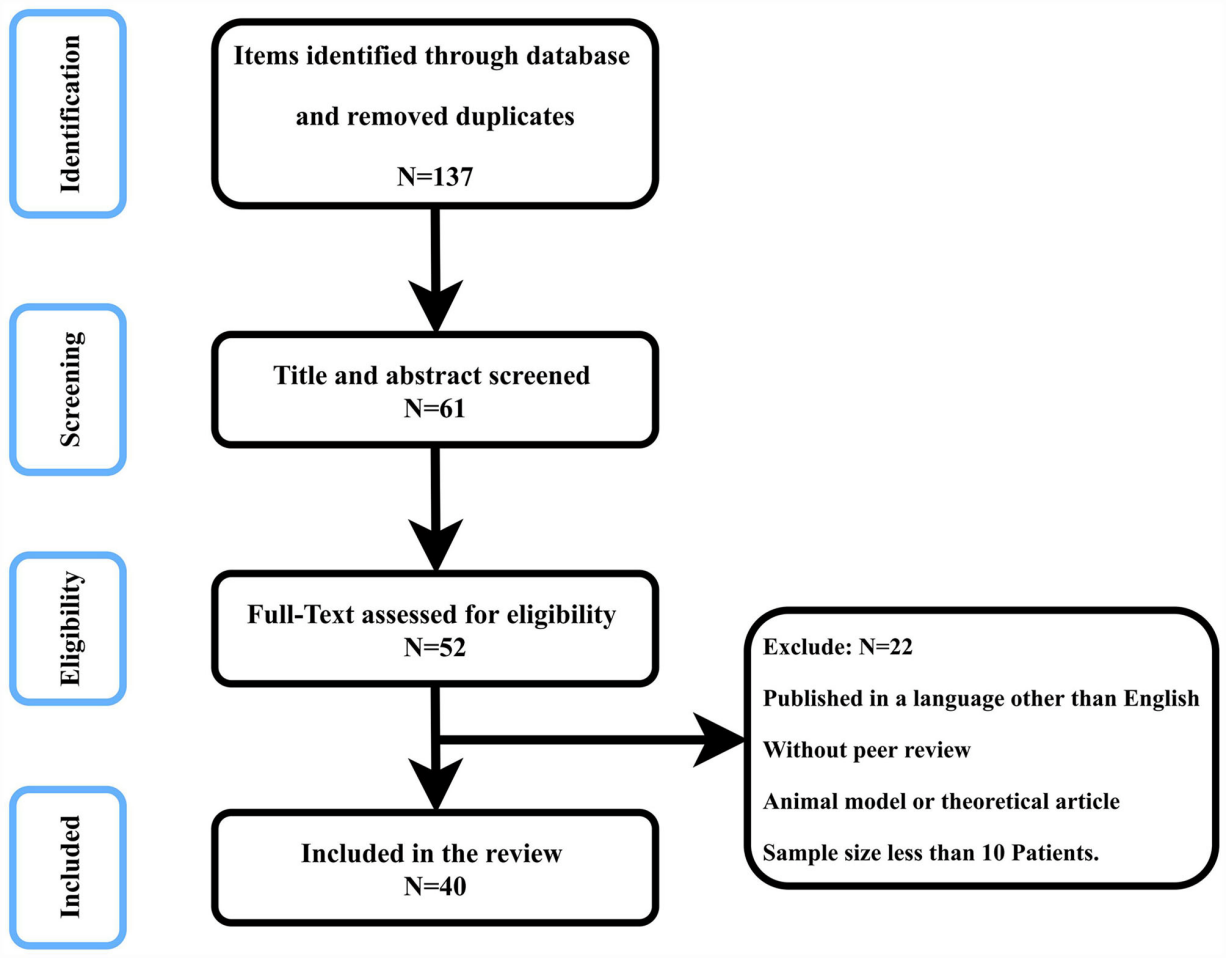
Flowchart of research selection.

**Figure 2:**
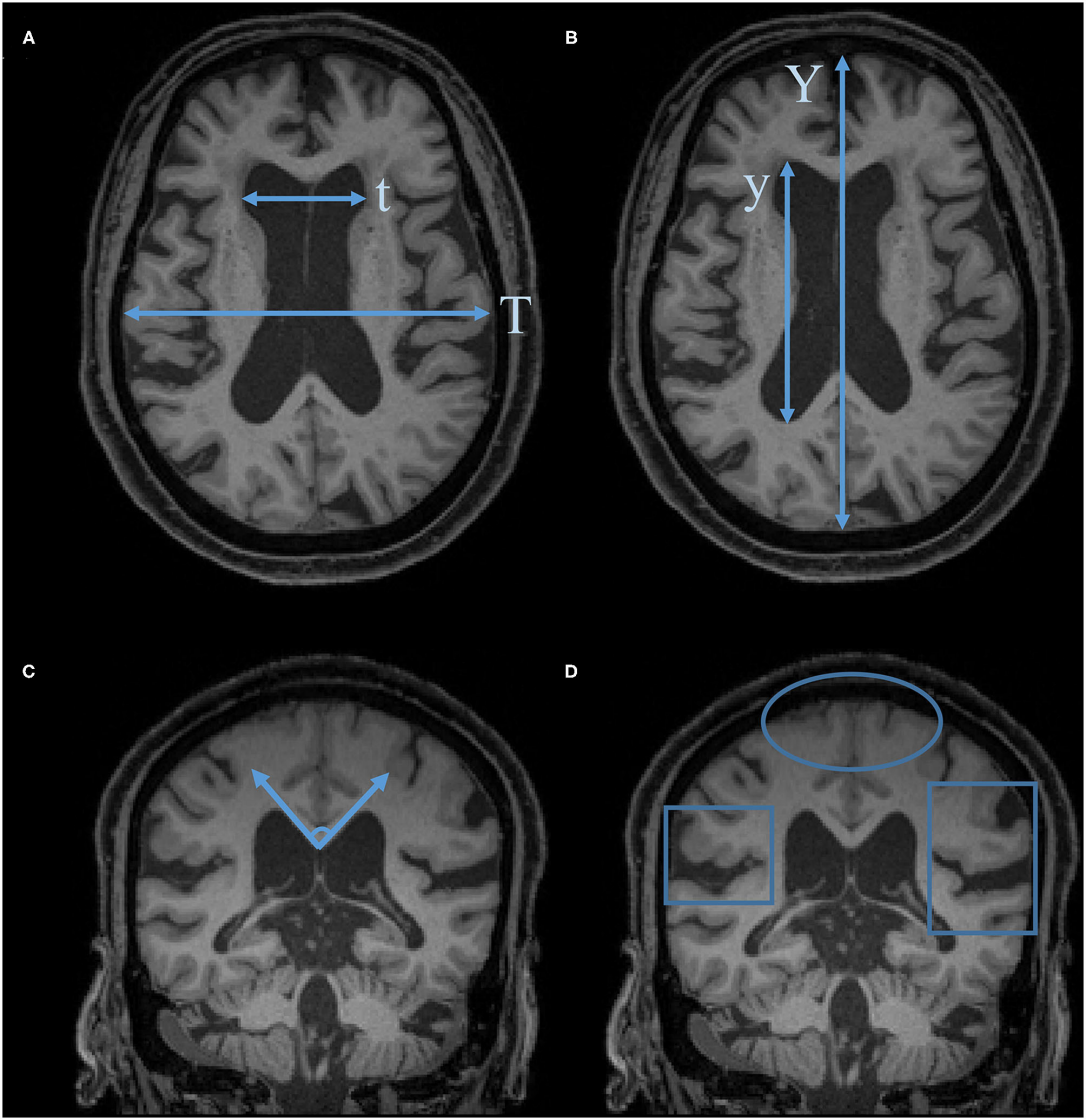
Characteristic imaging findings of NPH. **(A)** Evans Index (EI) = t/T. **(B)** Anteroposterior diameter of the lateral ventricle index (ALVI) = y/Y. **(C)** Callosal angle. **(D)** Disproportionately enlarged subarachnoid space hydrocephalus (DESH): Sylvian fissure enlargement and tight high-convexity effacement are present.

Figure 1 is a flowchart of study selection, and Figure 2 is the characteristic imaging findings of NPH. This review selected 40 references, of which 11 references provided the background introduction to NPH and EI, 7 referred to the measurement of the EI, 5 to EI threshold setting, 3 to the correlation between EI and VV, 10 to the clinical value of EI, and 4 to alternative indices to EI.

## Discussion

### Measurement Methods

On one hand, EI measurements are affected by the different imaging planes and angles. The following two data points are needed to calculate EI: the maximal width of the frontal horns and the maximum inner diameter of the skull. These two datasets can be easily measured using encephalography, as only one image is needed to perform both measurements. However, brain computed tomography produces numerous cross-sectional images, and the maximal width of the frontal horns and the maximum inner diameter of the skull may appear on the same or different images (Toma et al., 2011). Therefore, some researchers measured the inner diameter of the skull in the same plane as the maximal width of the frontal horns (Del et al., 2018), while others calculated the maximal width separately (Ambarki et al., 2010). The EI results calculated for different planes and angles have been shown to differ. Therefore, to systematically evaluate the changes in EI of NPH patients, the plane and angle used for EI calculation every time are strictly consistent. However, this is difficult to perform in clinical practice (Toma et al., 2011). More importantly, there is currently no unified standard to specify which plane and angle should be used for standardized EI measurements. The comparison of EI results using different planes and angles can lead to differences, which may be due to the different measurement methods rather than changes in the ventricle (Ryska et al., 2021). Therefore, standardized and unified imaging planes and angles are very important for the measurement of EI.

On the other hand, for NPH patients, EI measurement is a fast and highly reproducible indirect method for assessing VV (Bao et al., 2016; Liu et al., 2020). VV measurement methods can be direct and indirect (Ambarki et al., 2010). Direct VV measurement is achieved by segmenting the ventricle, the process of specifically marking the ventricle structure in image research (Huff et al., 2019). Direct VV measurements can be further subdivided into manual, semi-automatic, and automatic measurements (Cherukuri et al., 2018). The manual measurement is very time-consuming, subjective, and not highly reproducible and requires the operator to have professional knowledge of ventricular anatomy (Dubost et al., 2020). The automatic measurement overcomes the above shortcomings but needs to rely on special software, and the measurement results are not necessarily accurate (Kempton et al., 2011; Curra et al., 2019). Finally, the semi-automatic measurement is based on automatic measurement, with an operator making manual adjustments (Ntiri et al., 2021). In contrast, EI provides an indirect linear measurement method for the VV. Its measurement is very simple and fast, does not rely on software (Ambarki et al., 2010), and has excellent intra-and inter-observer reliability (Bao et al., 2016; Brix et al., 2017).

Therefore, the measurement of EI is affected by different imaging planes and angles. However, under a consistent imaging protocol, EI measurements are not only simple and rapid but also highly reproducible.

### Threshold Setting

Studies have shown that ventricle size is related to age and sex (Crook et al., 2020; He et al., 2020). Therefore, as an indirect measurement method of the VV, EI measurements are also affected by age and sex. Several recent studies have shown that in healthy elderly, when the EI threshold for judging whether ventricular enlargement is set to 0.3, >20% of healthy elderly individuals have ventricular enlargement (Yamada et al., 2015; Brix et al., 2017; Jaraj et al., 2017a). More importantly, there are also sex differences in the EI among the elderly. A study including 3193 axial computed tomography scans showed that men have a higher EI than women (Curra et al., 2019). Therefore, some researchers have set different EI thresholds for judging ventricular enlargement according to age and sex (Brix et al., 2017).

A study including 10 shunt-responsive NPH patients indicated that EI is not an ideal method for estimating the VV in NPH patients and questioned the use of EI alone as a marker of enlarged ventricles (Ambarki et al., 2010). However, another study including 34 probable idiopathic NPH patients, 34 Alzheimer's disease (AD) patients, and 34 healthy controls, showed that when using a combination of EI and callosal angle (threshold EI >0.30 and callosal angle <90°), the accuracy for distinguishing between NPH and AD patients was 96%, with a sensitivity of 97%, and a specificity of 94% (Ishii et al., 2008). Another study, including 36 shunt-responsive NPH patients, 34 AD patients, and 36 healthy controls, showed that the EI (at a threshold of >0.32) and callosal angle can be used as a screening tool to help distinguish NPH patients from non-NPH patients (Miskin et al., 2017).

### Correlation With VV

The EI is used as an imaging biomarker of NPH to indirectly assess the VV (Liu et al., 2020). A study including 23 definite iNPH patients and 62 healthy elderly volunteers, showed that the correlation between EI and VV and relative VV (RVV) was 0.843 and 0.840, respectively. Another study, including 20 patients with large ventricles and 46 healthy elderly subjects, showed a correlation between EI and VV and RVV of 0.94 and 0.95, respectively. These studies proved that the EI has an excellent correlation with VV but that the two reflect different properties; while the EI is calculated using the measurements from a plane, the ventricle is a three-dimensional structure (Ambarki et al., 2010; He et al., 2020).

However, a study including 10 shunt-responsive NPH patients showed that the correlation between EI and VV and RVV was 0.619 and 0.498, respectively, whereas another study including 36 iNPH patients showed that the correlation between EI and VV was 0.62. Based on these studies, the correlation between EI and VV is not good, and it is, therefore, not an ideal method for evaluating the VV in NPH patients (Toma et al., 2011; Bao et al., 2016).

### Clinical Values

EI is an important radiological change preceding symptoms in patients (Engel et al., 2018). Studies have shown that iNPH follows a spectrum of disease development, and radiological manifestations precede clinical symptoms (Iseki et al., 2014). Therefore, radiological manifestations may be an early sign of the disease (Jaraj et al., 2017b).

The EI is a screening tool for patients with NPH. A recent systematic review and meta-analysis on the application of EI in NPH patients indicated that the EI should be used as a screening tool for ventricular enlargement in NPH patients (Park et al., 2021). More importantly, since NPH is a treatable disease, early diagnosis and treatment can increase the probability of a good prognosis (Jaraj et al., 2017b). For screening purposes, performing fine ventricular structure analysis is time-consuming and labor-intensive (Miskin et al., 2017). In contrast, the use of simple linear measurements for screening can effectively improve time and cost (Bao et al., 2016).

It is difficult to characterize the gait and cognition of patients with NPH. VV measurements can better indicate the current and future gait and cognitive status of NPH patients than the EI (Crook et al., 2020), and no significant relationship between the EI and impaired cognition or gait has been described (Lilja-Lund et al., 2020). More importantly, a study including 36 iNPH patients who responded to CSF drainage and subsequently underwent ventriculoperitoneal shunt surgery showed that higher EI is a predictor of long-term cognitive improvement in NPH patients after surgery; however, there is no appropriate EI threshold to help clinicians accurately predict the surgical effect on NPH patients. Nevertheless, in said study, the authors assessed the long-term cognitive subjective outcomes after shunt surgery through telephone interviews (Subramanian et al., 2021). Another study included 314 non-disabled, stroke-free, individuals aged ≥60 years showed a significant and nearly linear inverse relationship between the EI and the Montreal Cognitive Assessment score, providing evidence of the utility of the EI in assessing cognitive performance (Del et al., 2018).

Changes in the size of the brain ventricles in patients with NPH after surgery are usually not detected by measuring the EI (Virhammar et al., 2018). Studies have shown that the VV of patients with NPH decreases after surgery. However, even if the clinical symptoms of NPH patients improve after surgery, the EI remains unchanged even during the entire follow-up process after surgery (Yamada et al., 2019). The clinical improvement in NPH patients after surgery is related to a decrease in ventricle size. Thus, to assess the changes in ventricle size in patients with NPH after surgery, VV measurements are more accurate than the EI measurements (Neikter et al., 2020).

### Alternatives to EI

On the one hand, the enlargement of the ventricles of patients with NPH, especially the frontal angle, has been shown to follow the z-axis direction instead of the x-axis direction. Thus, the z-EI (z-EI) is a representative index of the expansion of the frontal angles of the ventricles in the z-axis direction. The CSF drainage test (tap test) is useful for diagnosing iNPH and predicting the therapeutic effect of shunt intervention. A study, including 24 tap test-positive iNPH patients, 25 patients with no response to the tap test, and 23 healthy controls, showed that the relationship between the z-EI and NPH patients' response to the tap test was the most significant. In this study, the iNPH grading scale and a quantitative examination of gait and cognitive function were used to assess the improvement of symptoms before the CSF tap test and 1 and 4 days after the test (Yamada et al., 2015). Subsequent studies have shown that the expansion of the lateral ventricle toward the z-axis (z-EI) is a common parameter for distinguishing NPH from AD (Yamada et al., 2016). More importantly, in the follow-up process of NPH patients after surgery, the z-EI continued to change gradually changing, while the EI did not change (Yamada et al., 2019). Therefore, in the 2021 NPH guidelines, even an EI <0.30, in case of the presence of other indicators of an expanded inferior horn of the lateral ventricle, such as a z-EI >0.42, the diagnosis of possible NPH is acceptable (Nakajima et al., 2021).

On the other hand, the selection of different scan baselines and planes affects the measurement of EI. The anteroposterior diameter of the lateral ventricle index (ALVI) (He et al., 2020) is defined as the ratio of the lateral ventricle anteroposterior diameter measurement to the maximal width of the anteroposterior inner diameter of the skull (along the cerebral falx) in the same plane. Unlike the EI, ALVI measurements do not require the operator to measure from several consecutive axial slices to determine the maximum diameter. Therefore, the ALVI measurement method is simpler and clearer than the EI measurement method, thereby reducing the measurement deviation. More importantly, the threshold setting of ALVI >0.5 is more effective in assessing ventricular enlargement than an EI >0.30.

## Conclusion and Perspective

The measurement of EI is affected by different imaging planes and angles, and its normal range varies depending on both age and sex. Furthermore, the correlation between the EI and VV and its clinical significance is controversial. However, in several versions of the NPH guidelines, it is accepted that an EI >0.30 served to assess the ventricle enlargement. Clinically, the EI is the most commonly used indirect parameter for assessing the condition of the ventricle. This is explained because measuring the EI is simple, fast, and robust and does not require any specialized software or even knowledge of anatomy, whereas the direct measurement of the VV is time-consuming, laborious, and difficult to perform in large samples.

In the future, a unified agreement for the EI measurements needs to be met. Moreover, the EI threshold needs adjustments according to different purposes, and its clinical value needs corroboration.

## Author Contributions

XZ conceived and drafted the manuscript. JX reviewed the final version and made necessary changes. All authors contributed to the manuscript and approved the submitted version.

## Funding

This study was supported in part by the National Natural Science Foundation of China (grant number 82171913).

## Conflict of Interest

The authors declare that the research was conducted in the absence of any commercial or financial relationships that could be construed as a potential conflict of interest.

## Publisher's Note

All claims expressed in this article are solely those of the authors and do not necessarily represent those of their affiliated organizations, or those of the publisher, the editors and the reviewers. Any product that may be evaluated in this article, or claim that may be made by its manufacturer, is not guaranteed or endorsed by the publisher.
